# Combined simultaneous embolization of the portal vein and hepatic vein (double vein embolization) – a technical note about embolization sequence

**DOI:** 10.1186/s42155-021-00230-w

**Published:** 2021-05-26

**Authors:** Arash Najafi, Erik Schadde, Christoph A. Binkert

**Affiliations:** 1 Department of Radiology and Nuclear Medicine, Kantonsspital Winterthur, Brauerstrasse 15, 8401 Winterthur, Switzerland; 2 Department of Surgery, Kantonsspital Winterthur, Winterthur, Switzerland

**Keywords:** Portal vein, Hepatic vein, Embolization, Future liver remnant

## Abstract

**Background:**

Simultaneous portal vein embolization (PVE) and hepatic vein embolization (HVE) has been shown to be feasible, safe and lead to a faster growth of future liver remnant (FLR) than PVE alone. The objective of this study is to highlight different technical aspects as well as importance of embolization order.

**Materials and methods:**

Seven patients were treated with simultaneous PVE and HVE. In three cases, HVE was performed first followed by PVE and in four cases the other way around. Portal vein branches were embolized using Glubran-Lipiodol mixture in all cases. Hepatic veins were embolized using Amplatzer II plugs sized 8–20 mm. Specific consideration was given to depth of glue penetration in the portal vein defined by visible branch order on the treated side.

**Results:**

Six of seven patients were discharged home the same day. One patient with infected tumor necrosis died of liver failure 40 days later, otherwise there were no periprocedural clinical complications. Median glue penetration was to the 5th order (4th – 5th) when PVE was performed first and 3rd order (2nd - 4th) when PVE was performed after HVE. In one PVE first case, glue spillage was seen due to marked reduced flow in the right portal vein. There was sufficient FLR growth for subsequent surgical resection in the remaining six patients.

**Conclusion:**

PVE should be performed prior to HVE because the reduced flow in the portal vein after HVE leads to less deep glue penetration with presumably increased risk of contralateral spillage.

## Introduction

Liver resection is the first-line treatment for many primary and secondary liver malignancies, but can be associated with significant perioperative morbidity and mortality (van Lienden et al. 2013), with the main reason being inadequate volume of the future liver remnant (FLR) leading to post-hepatectomy liver failure. Several techniques have been developed to induce hypertrophy of the FLR, thereby increasing the likelihood of resectability and reducing the risk of post-operative complications (Madoff et al. 2020).

Recently, combined simultaneous embolization of portal and hepatic veins has been described and first studies show it to be a safe and feasible technique with faster growth rates than PVE alone (Guiu et al. 2016; Guiu et al. 2017; Kobayashi et al. 2020). As previously for PVE, several techniques with different embolization materials have been developed for HVE. The liver venous deprivation technique (Guiu et al. 2016) aims to prevent formation of distal venous-venous collaterals after hepatic vein embolization. Thus, the hepatic veins are punctured percutaneously and plugs are used for central vein occlusion with subsequent application of glue to embolize peripheral venous branches. The reported results are very promising; however there is a potential danger of liquid agent embolization to the lungs. Therefore, most centers have adapted the transjugular approach with embolization of the hepatic veins by means of multiple plug insertion both distally and proximally. This approach is popularized under the term double vein embolization.

Initially, our center performed HVE first for sake of better visibility of the hepatic veins (no overlay of glue in the portal veins), but then switched to a PVE-first approach. To the best of our knowledge, no study has thus far assessed the impact of the order of embolization of the portal vein respectively hepatic vein on the embolization procedure.

## Materials & methods

This retrospective, single center study was conducted with institutional review board approval and written, informed patient consent.

### Study population

Between July 2017 and April 2020, seven patients (four males, three females, age 30–82 years) in whom PVE alone was deemed probably insufficient were treated at our institute with the double vein embolization technique. Four patients had hepatic colon cancer metastases while the other three patients had metastases from small bowel neuroendocrine tumor, solitary fibrous tumor, and large intrahepatic cholangiocarcinoma, respectively. Three patients had prior atypical wedge resections of the left liver in the context of a two-stage hepatectomy. Patient demographics are summarized in Table 1.

**Table 1:** Patient demographics

Case Number	Age (years)	Sex	Malignancy	Prior Intervention / Surgery
1	47	M	Neuroendocrine tumor of small bowel	None
2	67	F	Colon Cancer	None
3	65	M	Colon Cancer	Atypical wedge-resections segments II, III
4	72	F	Colon Cancer	None
5	57	M	Abdominal solitary fibrous tumor	Atypical wedge-resections segments III, IVb
6	30	M	Colon Cancer	Atypical wedge-resections segments I, II, III, IV
7	82	F	Large intrahepatic cholangiocarcinoma	Percutaneous transhepatic cholangio-drainage

### Procedure details

Coagulation parameters were tested 24 h before the procedure, ensuring an INR < 1.5 and platelet count of at least 50,000/mm^3^, according to recent guidelines from the Society of Interventional Radiology (Patel et al. 2019). Anticoagulation medication was stopped a few days prior. All interventions were performed under conscious sedation by means of midazolam and fentanyl +/− propofol in cases where patients started to experience more than moderate amount of pain. All interventions were performed by two experienced interventional radiologists with more than 10 years’ experience in PVE.

Access to the ipsilateral, right portal venous system was gained through a peripheral right portal branch under ultrasound-guidance using an AccuStick Introducer System (Boston Scientific), followed by portal venography. Anterior and posterior segmental portal branches of the right hemi-liver were selectively catheterized separately by using a reverse-curved catheter (e.g. 5F SOS Omni) and a 2.7 F Microcatheter (Progreat Micro Catheter System). Glubran 2 (GEM) was used as the liquid embolic with a Lipiodol (Guerbet) mixture of 1:1 or 1:2. In one case micro-coils were additionally deployed in a small portal branch. After embolization, the puncture tract was closed by injecting glue or gelfoam while retracting the catheter/sheath.

Access to the right hepatic vein was obtained using a transjugular approach with introduction of a long 6-7F sheath, followed by a hepatic venogram. Both peripheral and central branches of the right hepatic vein were occluded using several Amplatzer type II plugs of 8–20 mm diameter with minimal 10 mm distance to the inferior vena cava junction to reserve space for surgical transection.

In the initial three cases, HVE was performed first followed by PVE while in the later four cases PVE was done before HVE. Percentage of standardized FLR (sFLR) was calculated on imaging prior to intervention and on the first imaging post intervention. Degree of hypertrophy is given as absolute percentage value of the difference between pre- and post-interventional sFLR.

### Glue penetration

Penetration depth of glue was retrospectively assessed by a board-certified radiologist with 2 years’ experience in interventional radiology by looking at post-interventional visibility of the most distal branch order where glue had entered on fluoroscopic images. This was taken as surrogate of portal venous flow during PVE. The right and left portal veins were given branch order 0 and the first subsequent branch or bifurcation order 1 etc. This definition derived from the fact that catheter placement was usually in first-order branches of the portal veins. Additionally, spillage of glue to the contralateral side was documented.

## Results

During all procedures, pain was well tolerated under conscious propofol sedation and patients were discharged home the same day. One patient with cholangiocarcinoma died on day 40 as a result of septic shock due to infected tumor necrosis after the intervention. She had a necrotic tumor core prior to the intervention and dilated biliary ducts that had been drained percutaneously. She underwent embolization as an inpatient assuming the drainages were sufficient. She died from progressive liver failure.

In the other six patients, there were no post-procedural clinical complications, specifically no post-embolization syndrome that could not be addressed with analgesia and antipyretic medications at home, bleeding, infection, or abscess formation. There were no re-admissions.

**Fig. 1):**
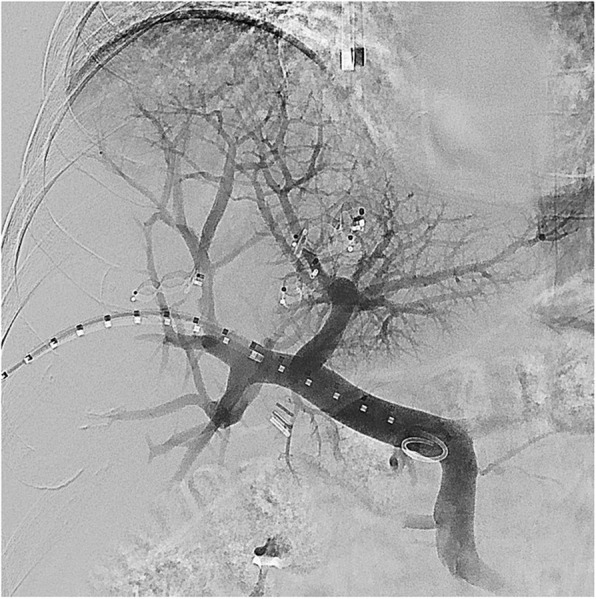
Portal venogram from the main portal vein after plug embolization of the right and middle hepatic vein with 2 plugs each. Obvious slower flow in the right portal branches with depiction of 2nd to 3rd order branches while 4th to 5th order branches are visible on the left side with parenchymal blush. Blushing of the liver dome on the right is through shunting from the left side

**Fig. 2:**
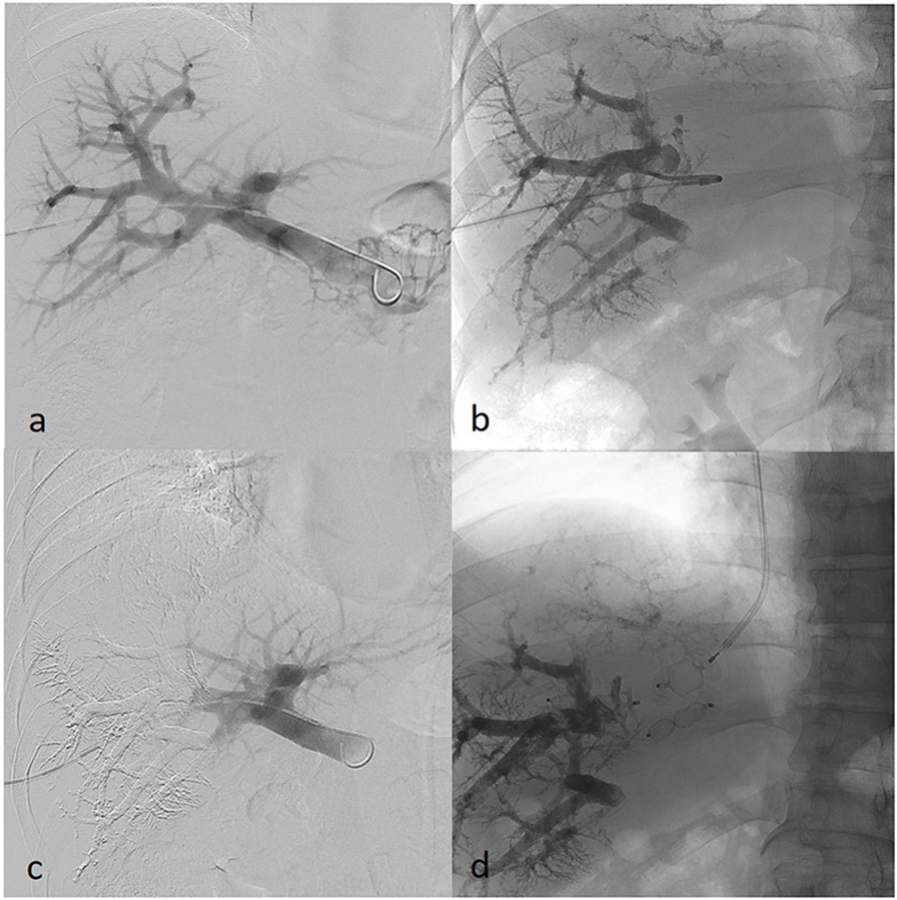
**a-d** Combined embolization of the portal vein and then right hepatic vein. **a** portal venogram before embolization. **b** embolization of the right portal branches with Glubran 2 and Lipiodol with casting of fifth order branches. **c** portal venogram after embolization. **d** transjugular hepatic plug insertion using 12 mm and 14 mm diameter Amplatzer type II plugs

**Fig. 3:**
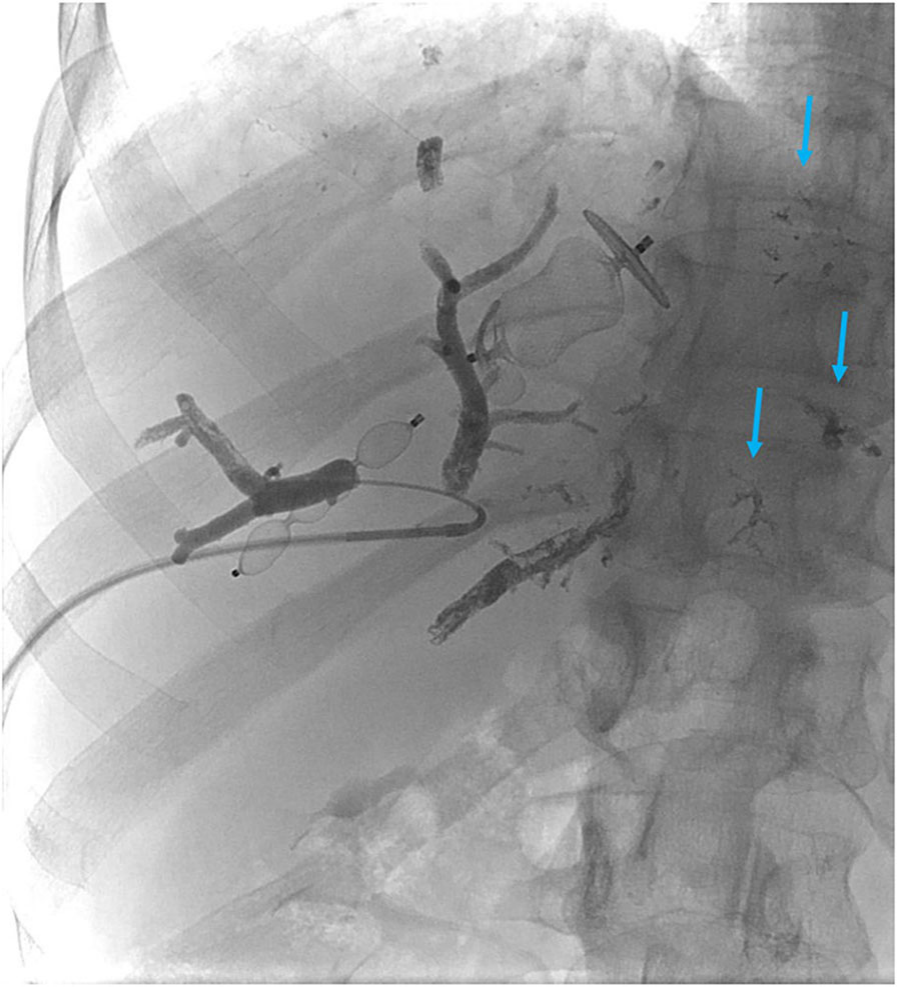
Embolization of the portal vein after right hepatic vein embolization. Amplatzer plugs already inserted. Only 3rd order branches were filled with the Glubran 2 - Lipiodol mixture. Because of the very slow flow in the right portal vein some glue spilled to the other side (blue arrows) despite a selective microcatheter position

Number and size of used plugs and amount of glue used in each case is summarized in Table 2. Median glue penetration depth was to the 3rd portal vein branch order in cases where HVE was performed first (2nd - 4th) and 5th branch order (4th - 5th) in cases where PVE was performed first (Figs. 1 and 2). Glue spillage to the contralateral side was noted in one patient in whom HVE was performed first (Fig. 3).

**Table 2:** Technical details about embolization material, order of embolization and glue penetration depth

Case Number	Embolization Hepatic Veins	Embolization Right Portal Vein	Portal Vein Branch Order
1	First: Right hepatic vein with 2 Amplatzer plugs type II (12 and 20 mm)	Second: 2 ml Glubran-Lipiodol 1:2 and 6 micro-coils	3
2	First: Right hepatic vein with 2 Amplatzer plugs (10 mm and 14 mm) and middle hepatic vein with 2 plugs (8 mm and 12 mm)	Second: 6 ml Glubran-Lipiodol 1:1	2
3	First: Right hepatic vein with 3 Amplatzer plugs type II (10, 16, 20 mm)	Second: 16 ml Glubran-Lipiodol 1:1	4
4	Second: Accessory right inferior hepatic vein and right hepatic vein with 6 Amplatzer plugs type II (8–14 mm)	First: 10 ml Glubran-Lipiodol 1:1	4
5	Second: Accessory right inferior hepatic vein and right hepatic vein with 3 Amplatzer plugs type II (12–14 mm)	First: 10 ml Glubran-Lipiodol 1:1	5
6	Second: Accessory right inferior hepatic vein and right hepatic vein with 7 Amplatzer plugs type II (10–20 mm)	First: 9 ml Glubran-Lipiodol 1:2	5
7	Second: Middle and right hepatic vein with 7 Amplatzer plugs type II (8–20 mm)	First: 9 ml Glubran-Lipiodol 1:2	5

Sufficient growth of the sFLR was reached in six out of seven cases (%sFLR between 22 and 51% after 7–31 days) with subsequent surgical resection (Table 3). There were no major post-surgical complications (especially no post-hepatectomy liver failure) and 30-day surgical mortality was 0%.

**Table 3:** Percentage of standardized future liver remnant (%sFLR) pre and post embolization as well as degree of hypertrophy

Case Number	%sFLR before embolization	%sFLR after embolization	Degree of Hypertrophy
1	33%	51% (after 9 days)	18% (after 9 days)
2	21%	35% (after 7 days)	14% (after 7 days)
3	20%	37% (after 19 days)	17% (after 19 days)
4	25%	31% (after 7 days)	6% (after 7 days)
5	14%	22% (after 22 days)	8% (after 22 days)
6	18%	35% (after 31 days)	17% (after 31 days)
7	12%	18% (after 15 days)	6% (after 15 days)

## Conclusion

In case of embolization of the hepatic vein first, the flow in the portal veins is markedly reduced, preventing distal portal vein embolization and presumably increasing risk of non-target embolization and contralateral spillage. Hence, we strongly recommend performing PVE prior to HVE.

## Data Availability

The datasets generated and/or analysed during the current study are not publicly available due to restrictions by law regarding protection of patient data but are available from the corresponding author on reasonable request.

## Abbreviations

PVE: Portal Vein Embolization
HVE: Hepatic Vein Embolization
FLR: Future Liver Remnant
sFLR: Standardized Future Liver Remnant
